# Chasmogamy and entomophily in *Burmannia disticha* (Burmanniaceae)

**DOI:** 10.3389/fpls.2023.1237665

**Published:** 2023-08-23

**Authors:** Nikolay A. Vislobokov, Maxim S. Nuraliev

**Affiliations:** ^1^ Department of Higher Plants, Faculty of Biology, M.V. Lomonosov Moscow State University, Moscow, Russia; ^2^ Joint Russian-Vietnamese Tropical Scientific and Technological Center, Hanoi, Vietnam

**Keywords:** Bat Xat Nature Reserve, *Bombus*, Dioscoreales, pollen-ovule ratio, pollination, proboscis, Vietnam, zoophily

## Abstract

*Burmannia* shows a set of floral traits that suggest elaborate mechanisms of animal-mediated pollen transfer. These include flower coloration, septal nectaries and a long and narrow floral chamber. The stamens are synorganized with the common style restricting the entrance to the floral chamber, sometimes forming a gynostegium. Contrary to this apparent zoophilous floral syndrome, several species of *Burmannia* were reported to perform self-pollination via cleistogamy. Understanding of reproductive systems in *Burmannia* is complicated by scarcity of available results of direct observations on pollination process. Here we present data on pollination biology of *B. disticha* obtained during field investigations in Vietnam followed by laboratory analyses of ecologically important floral traits and the captured flower visitors. We found that the anthetic perianth is open, i.e. the flower is chasmogamous. The flowers are visited by various Diptera, Hymenoptera, Lepidoptera and Orthoptera. Of them, the bumblebees (*Bombus burmensis*), a bee (*Coelioxys* sp.) and some lepidopterans were revealed to carry pollen of *B. disticha*. Based on the amount of carried pollen, insect behavior during the visits and general knowledge on biology of these insect taxa, we concluded that the bumblebees act as the principal pollinators of *B. disticha*, whereas the lepidopterans are considered as its possible pollinators. We compared the lengths of proboscises of the captured insects to the depth of the floral chamber, and found that only the bumblebees and lepidopterans should be able to reach the nectar. Finally, we estimated the pollen-ovule ratio of *B. disticha* as 6.84, which is comparable to the ratio known in autogamous angiosperms. Based on its flower organization and pollination mechanism, we consider *B. disticha* an entomophilous and predominantly xenogamous species. Its gynostegium is likely an adaptation for pollen transfer by insects with long proboscises. At the same time, earlier investigations together with pollen-ovule ratio indicate that *B. disticha* possesses a labile pollination strategy, and autogamy sometimes occurs. Since *Burmannia* is one of the few angiosperm genera that comprise both mycoheterotrophic (achlorophyllous) and autotrophic (green) species, our study provides important evidence for reconstructions of ecological and morphological evolutionary pathways in relation to the mode of organic nutrition.

## Introduction

*Burmannia* L. is a genus of herbaceous plants which comprises about 60 species (Nuraliev et al., 2018; Li et al., 2020; Francis et al., 2021; Govaerts et al., 2022; Nuraliev et al., 2022). The genus possesses a pantropical distribution with some representatives extending into subtropical areas; about a half of its species are found in South, East and Southeast Asia (Jonker, 1938; Wood, 1983; Maas et al., 1986; Saunders, 1996; Zhang, 1999; Hsieh and Ohashi, 2000; Wu et al., 2010; Mesta et al., 2011; Tsukaya and Darnaedi, 2012; Merckx et al., 2013; Dang et al., 2015; Suetsugu and Sando, 2017). About one third of the species of *Burmannia* are fully mycoheterotrophic (lacking chlorophyll). The other species are photosynthetic, several of which are fully autotrophic, whereas the rest are presumably partially mycoheterotrophic (Jonker, 1938; Wood, 1983; Maas et al., 1986; Merckx et al., 2010; Merckx et al., 2013; Suetsugu et al., 2014). *Burmannia* therefore represents a convenient model for investigation of ecological traits in angiosperms with different modes of organic nutrition.

Inflorescence of *Burmannia* is a thyrsoid comprising two cincinni, which is transformed into a botryoid in some species via reduction of the lateral cymes to single flowers (Yudina et al., 2022). The flowers are bisexual, actinomorphic, tetracyclic, trimerous, with long and narrow floral tube. In most species, the floral tube bears prominent longitudinal wings. Tepal lobes are placed on the top of the floral tube. The inner tepal lobes are smaller than the outer ones, and in mature flowers of some species they are absent. Three sessile stamens are inserted on the inner surface of the floral tube close to its apex, opposite the inner tepals. The stamen connective is broad and usually appendaged, bearing two apical crests and a basal spur. The gynoecium is syncarpous, tricarpellate, with inferior ovary, supralocular septal nectaries and a filiform common style as long as the floral tube. The distalmost carpel parts are free and represent the three style branches terminating into stigmas. The stigmas alternate with the stamens. They are bent to the abaxial side so that they face the perianth and almost completely obstruct the orifice of the floral tube. Each stigma bears a transverse folding covered by papillae. The stamens are placed just below the stigmas, and the ventral surfaces of the connectives contact the apical part of the common style (Maas et al., 1986; Zhang, 1999; Caddick et al., 2000; Yudina et al., 2022). In some species (e.g. *B. chinensis* Gand., *B. disticha* L., *B. itoana* Makino, *B. lutescens* Becc. and *B. oblonga* Ridl.), the connectives are postgenitally fused to the common style forming a gynostegium, whereas in the other species (e.g. *B. championii* Thwaites, *B. coelestis* D.Don and *B. longifolia* Becc.) such a fusion does not take place, i.e. the gynostegium is not formed (Yudina et al., 2022).

Previous authors supposed occurrence of pollinator-independent autogamy (mostly via cleistogamy) in certain species of *Burmannia* (Ernst and Bernard, 1912; Schoch, 1920; Spitmann, 1975; Wood, 1983; Zhang, 1999; Zhang and Saunders, 2000) and entomophily in the others (Spitmann, 1975; Maas et al., 1986; Kato, 1996; Momose et al., 1998; Zhang, 1999). Available pieces of evidence that demonstrate a range of reproductive systems in *Burmannia* are briefly reviewed by Yudina et al. (2022). *Burmannia* almost uniformly shows a set of indirect indications in favor of cross-pollination via animal vectors, including complex floral construction, bright and contrasty floral coloration, presence of septal nectaries, papillose adaxial (inner) surface of the outer tepal lobes, and protandry (Spitmann, 1975; Maas et al., 1986; Zhang and Saunders, 2000; Yudina et al., 2022). At the same time, floral biology of *Burmannia* has never been investigated by means of experiments with artificial cross-pollination and flower isolation. Drawing conclusions on floral biology of the species of *Burmannia* is further complicated by the possibility of the presence of more than one reproductive system in a given species, as, for example, was supposed by Zhang (1999) for *B. itoana*.

Self-pollination was supposed for *B. biflora* L., *B. championii*, *B. disticha* and *B. lutescens* on the basis of floral morphology (Schoch, 1920; Fryxell, 1957; Wood, 1983). The stigmas of these species (as well as in *Burmannia* in general) are fan-shaped and strongly bent to the abaxial side, so that they obstruct the gaps between the neighboring anthers. In addition, the inner tepals of these species are described as being curved inward and obstructing the gaps between stigmas. It was believed that such a closed structure of flower should prevent cross-pollination (Schoch, 1920; Wood, 1983). Then, *B. capitata* Mart., *B. championii*, *B. lutescens* and *B. stuebelii* Hieron. & Schltr. were recognized as self-pollinated, because their pollen was observed to be released before the flower opened, and the stigmas of preanthetic flowers were covered with germinating pollen grains (Ernst and Bernard, 1912; Spitmann, 1975; Wood, 1983). In addition, Zhang (1999) described flowers of *B. disticha* and *B. itoana* observed in natural habitats to remain closed at least till the pollen is dispersed and ovules are fertilized; he therefore considered flowers of these species to be cleistogamous. Zhang (1999) traced the entire flowering process of *B. itoana* (transferred from a natural habitat to an isolated glass-covered pot), and observed the flowers to be permanently closed and the fruits and seeds to be set, which confirmed the cleistogamy. Zhang (1999) also noted several other species of the genus to perform cleistogamy, including *B. championii*, *B. coelestis*, *B. sphagnoides* Becc. On the other hand, Nuraliev et al. (2022) reported fully open fresh flowers in Vietnamese populations of *B. coelestis* and *B. itoana*.

Presence of starch in pollen grains of *B. chinensis*, *B. disticha* and *B. wallichii* Hook.f. was considered as an indication of autogamous mating system of these species (Zhang, 1999; Zhang and Saunders, 2000), because bees and flies usually pollinate plants with starchless pollen, whereas plants with starchy pollen grains are typically self-pollinated, wind-pollinated, or pollinated by butterflies or birds (Baker and Baker, 1979).

Apomixis was demonstrated for *B. coelestis*; in this species, pollen grains and embryo sacs consist of diploid cells (showing diplospory) and fertilization does not occur (Ernst, 1909). Apomixis was also supposed for *B. itoana* on the basis of genetic structure within its population inferred from the results of allozyme electrophoresis (Zhang, 1999). This is consistent with cleistogamy reported by Zhang (1999) for this species.

There are only a few reports of direct field observations of the anthesis in representatives of *Burmannia*. No floral visitors were detected during observations on *B. chinensis* and *B. wallichii* (Zhang, 1999; Zhang and Saunders, 2000), whereas flowers of *B. lutescens* were visited by Culicidae (mosquitoes), Diptera, which were regarded as its potential pollinators (Kato, 1996; Momose et al., 1998). In particular, Kato (1996) reported *B. lutescens* to be visited by mosquitoes from the genera *Armigeres* and *Culex*, whose proboscises correspond to the width of the openings between stigma and stamens and to the depth of the floral tube. These visits occurred at dawn and in the early evening.

In the present study, we investigated pollination biology of *Burmannia disticha*, a species previously considered to have cleistogamous flowers, and at the same time possessing indications of entomophily. In particular, we report results of our direct field observations of the plants *in situ* during the flowering period and the data on floral visitors of this species, including the diversity of pollen found on their bodies. Finally, we estimate pollen-ovule ratio for *B. disticha*, representing the first investigation of this index in Burmanniaceae.

## Materials and methods

### Study site and characteristics of the species under study

Field investigation of *Burmannia disticha* was performed in northern Vietnam (Lao Cai province, Bat Xat district, Bat Xat Nature Reserve); the general information on Bat Xat Nature Reserve is provided by Bui et al. (2020). The study site is located around the point 22° 37.539’ N, 103° 37.658’ E. Voucher herbarium specimens were collected under the collector’s number *Vislobokov 19039* and deposited at MW (barcode MW0757926) and at HN. The field investigations took place during 4–13 June 2019. The time of sunrise was 5:20 and time of sunset was 18:48 at the study site during this period, as estimated using the Solar Calculator available at the ESRL (2023) website.

**Figure 1:**
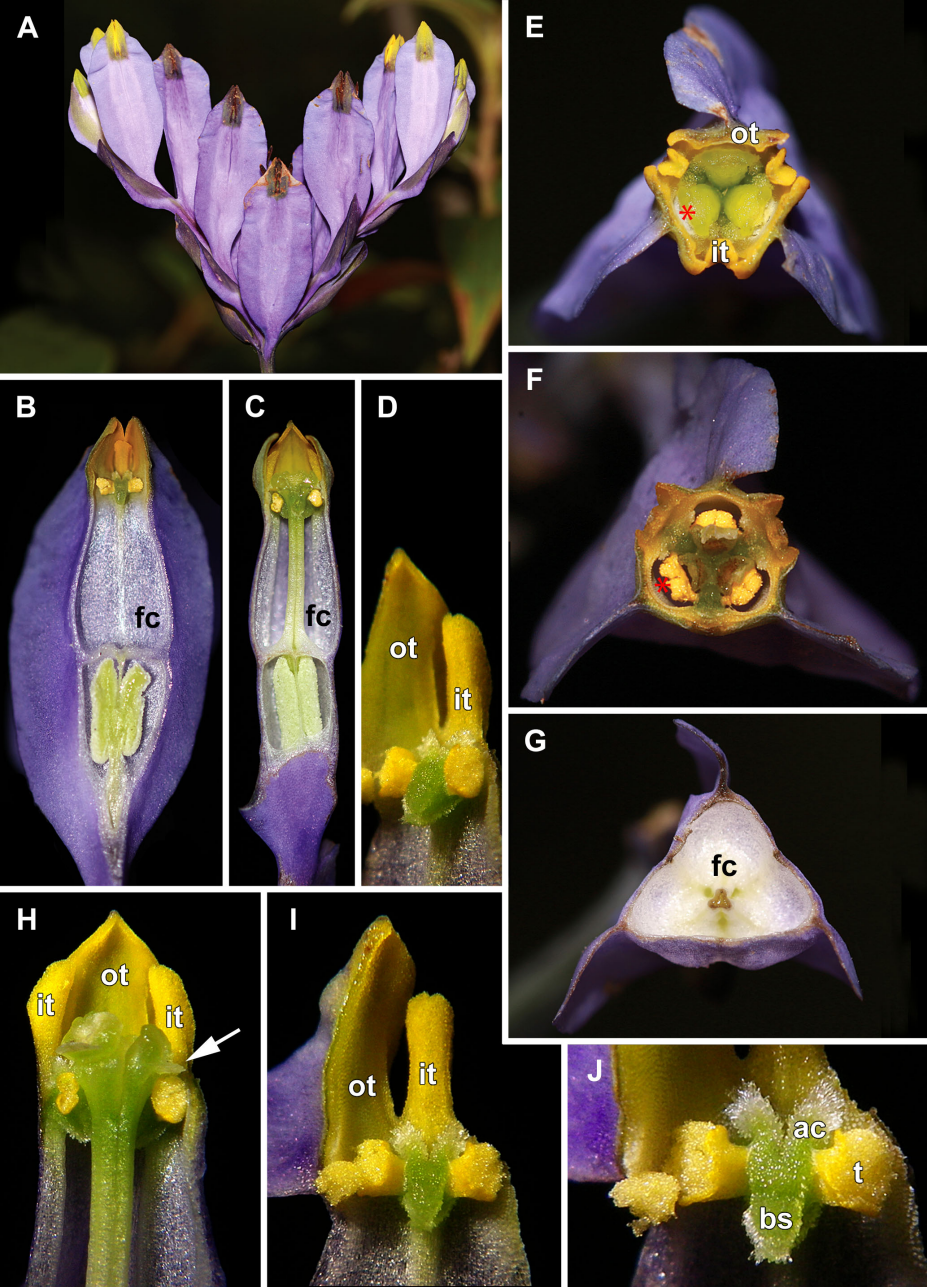
Flowers of *Burmannia disticha*. **(A)** Inflorescence (side view), showing flowers at different stages of anthesis. **(B, C)** Longitudial sections of flowers; section without common style in **(B)**. **(D)** Stamen (semi-side view). **(E)** Flower with distal portions of tepal lobes removed (top view). **(F)** Cross section of flower at level of anthers. **(G)** Cross-section of flower at level of floral tube. **(H)** Artificially opened flower showing arrangement of stamens, common style and stigmas. **(I)** Outer tepal lobe, inner tepal lobe and stamen. **(J)** Stamen, (front view). ac, apical crest of stamen connective; bs, basal spur of stamen connective; fc, floral chamber; it, inner tepal lobe; ot, outer tepal lobe; t, anther theca; arrow denotes membranous part of transversally folded stigma (insects get access to the floral chamber by turning down this membranous part); asterisk denotes the place of visitors’ access to floral chamber.

**Figure 2:**
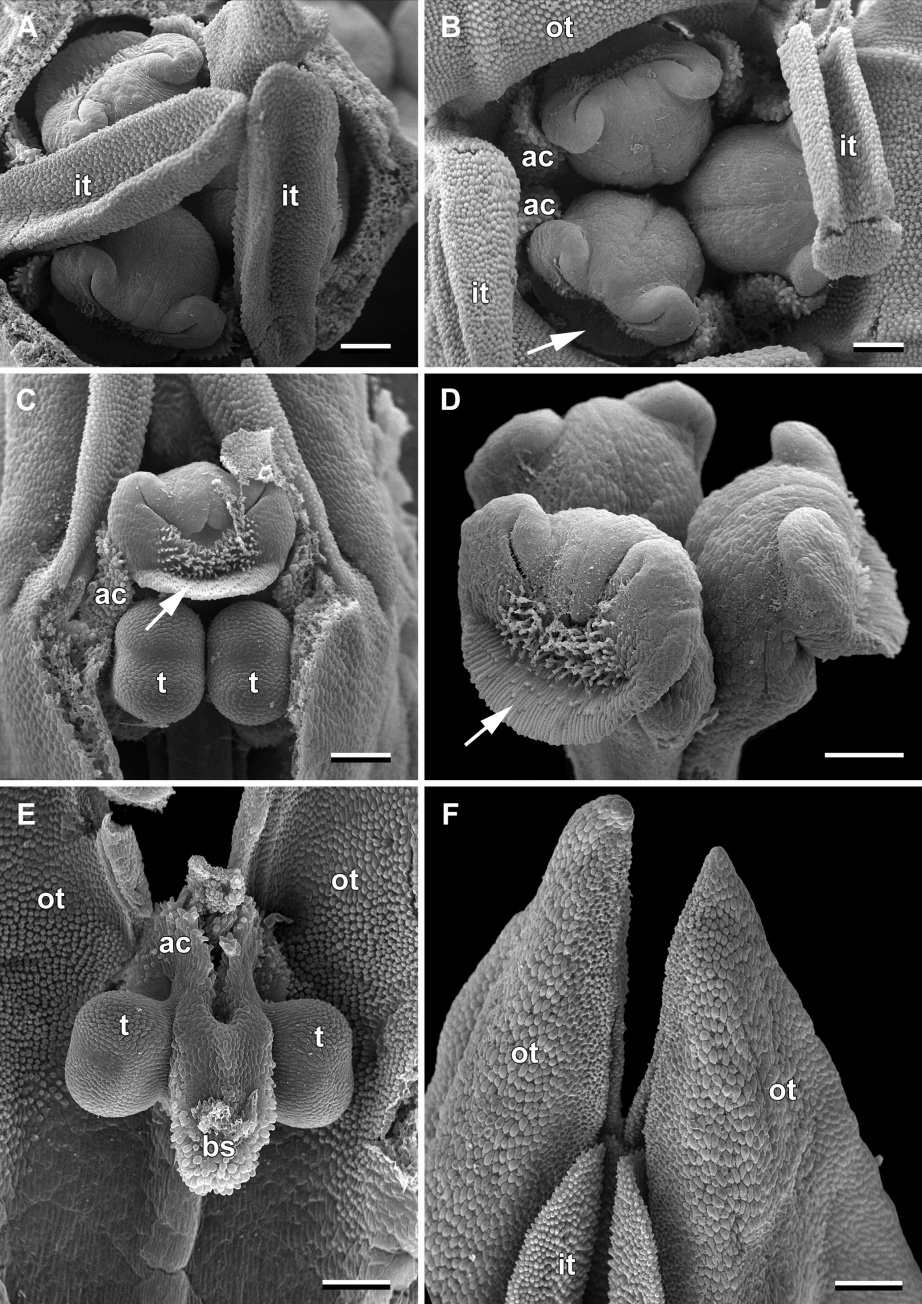
Flower buds of *Burmannia disticha* (SEM images). **(A)** Flower bud with outer tepal lobes removed (top view). **(B)** Flower bud with inner tepal lobes artificially bent aside (top view). **(C)** Flower bud with part of floral tube and one outer tepal lobe removed (side view). **(D)** Style branches (oblique view). **(E)** Stamen (view from adaxial side of floral tube). **(F)** Tepal lobes (side view). ac, apical crest of stamen connective; bs, basal spur of stamen connective; it, inner tepal lobe; ot, outer tepal lobe; t, anther theca; arrow denotes membranous part of transversally folded stigma (insects get access to the floral chamber by turning down this membranous part). Scale bars = 300 mm.

Flowers of *Burmannia disticha* (illustrated in **Figures 1**, **2**) are most often uniformly blue to violet outside except for yellow or greenish perianth lobes (Jonker, 1938; Jonker, 1948; Zhang, 1999). The floral tube is 3–8 mm long. The perianth wings are conspicuous, 1–3.5 mm wide, running from the pedicel along the ovary and floral tube to the outer tepal lobes, continuing into abaxial crest-like keels of the outer tepal lobes (Wu et al., 2010). The inner tepal lobes are well developed in mature flowers. The stamen connectives are postgenitally fused to the common style to form a gynostegium. The stigmas are placed right above the thecae and occlude the flower entrance (Yudina et al., 2022).

### Field observations

For observations of successive stages of anthesis, inflorescences were labeled individually and checked daily. Thirty-one inflorescences were monitored in total (each inflorescence on a separate above-ground shoot). Observations of floral visitors were performed during day time and night time on 4–13 June 2019. Observations were performed in a series of overlapping time intervals, i.e., different time intervals were covered on different days, so that altogether they cover all the day periods except for 2:00–6:00. The total observation time was 40 hours. Visual observations were performed as well as video recording and photography using HC-VX1EE-K (Panasonic, Japan), GZ-R495BEU (JVC, Japan) and EOS 70D (Canon, Japan) digital cameras. Insects visiting flowers of *B. disticha* were captured by a net, killed by ethyl acetate fumes, and fixed individually in 95% ethanol. The records of visits were based on captured visitors, direct observations and video records. Terminology related to plant reproduction follows Cardoso et al. (2018).

### Light microscopy

For analysis of pollen grains attached to the insect bodies, in Moscow State University, each insect was washed individually in 95% alcohol. Several drops of glycerol were added to the washing, after which the washing was mixed and centrifuged for 5 min at 3000 rpm. After that, 50 µl of sediment was taken from each sample, placed on a slide and investigated using a Micromed-2 light microscope (Ningbo Sheng Heng Optics & Electronics, China). All pollen grains were counted in each sample; pollen of *B. disticha* and pollen of all the other seed plants was recorded separately. Fourteen insects were sampled in total. Identification of pollen grains of *B. disticha* was performed by their comparison against pollen from anthers of this species. Since *B. disticha* is the only species of Burmanniaceae observed in the study area, misidentification of its pollen was unlikely.

Counting of pollen and ovules in flowers of *B. disticha* was performed in Moscow State University, using flowers with undehisced thecae fixed in 70% ethanol. Thecae and ovaries were dissected in 70% ethanol under an Olympus SZX7 (Olympus Corp., Japan) stereomicroscope. All pollen grains from one theca were placed on a slide and counted. All ovules from one placenta were placed on a slide and counted. Slides with pollen and ovules were examined using a Micromed-2 light microscope. Six thecae and six placentas from six different flowers (each flower taken from a separate plant) were investigated in total. Pollen-ovule ratio for the species was estimated by the following formula: P/O = 6*p*/3*o*, where *p* is the mean number of pollen grains in one theca and *o* is the mean number of ovules in one placenta.

For estimation of length of floral tube, 10 flowers were dissected in 70% ethanol under an Olympus SZX7 stereomicroscope and measured by a ruler and a vernier caliper. Similarly, length of proboscises of insects (fixed and stored in 95% ethanol) were measured by a ruler under an Olympus SZX7 stereomicroscope.

### Scanning electron microscopy

Scanning electron microscopy (SEM) was employed to investigate the mutual arrangement of organs inside the floral tube, and also to describe the fine characters of shape and surface of flower organs. The flowers fixed in 70% ethanol were dissected under an Olympus SZX7 (Olympus Corp., Japan) stereomicroscope, dehydrated through 80% ethanol, 96% ethanol and a mixture of ethanol (96%) and acetone (100%) at a 1:1 proportion followed by absolute acetone. The material was then critical-point dried in a Hitachi HCP-2 critical-point drier (Hitachi, Japan) using liquid carbon dioxide. Dried samples were mounted onto stubs using double-sided sticky tape, coated with gold using an Eiko IB-3 ion coater (Eiko Engineering Co. Ltd, Japan), and observed using a CamScan S2 SEM (CamScan, UK) at Moscow State University. Terminology for floral structure follows Yudina et al. (2022).

## Results

### Flowering dynamics

In June, massive flowering of *B. disticha* was recorded in Bat Xat Nature Reserve. In each inflorescence, the terminal flower opens first, followed by the two lateral flowers (i.e. the first flowers of the lateral cincinni) (**Figure 1A**). A flower of the next order opens when signs of senescence appear in the currently anthetic flower, or 1–2 days after that. Within a cincinnus, the lifespans of two adjacent flowers never overlap. Thus, there is normally a single blooming flower in each cincinnus at a given moment, and not more than two flowers blooming simultaneously in an inflorescence. A flower opens between 10:00 and noon (probably depending on weather). Tepal lobes are greenish yellow in flower bud becoming bright yellow right before flower opening. Tepal lobes are tightly appressed to each other in bud (**Figures 2A, F**); at flower opening they separate from each other and maintain the erect (upright) position during anthesis (**Figure 1H**). The inner tepal lobes are arranged close to the gaps between the neighboring style branches (**Figures 1B–E**). Anthers of all three stamens dehisce simultaneously at flower opening (**Figures 1F, H–J**, **2E**). No evidence of dichogamy was observed. The stamen connective possesses a pair of pronounced divergent apical crests and a thick pendent basal spur; all three appendages are distally covered by long papillae (**Figures 1D, H–J**, **2E**). The floral tubes of anthetic flowers were 5.3–8.1 mm long. Septal nectaries produce nectar, which is accumulated in the form of a film at the bottom of the floral chamber (**Figures 1B, C, G**). The stigma is fan-shaped, with a thin membranous transverse folding, covered with dense brush-like papillae (**Figures 2B–D**). In the anthetic flower, the stamens and stigmas are enclosed in the floral chamber which is hardly accessible from the outside: the orifice of the floral tube is obstructed by the apical crests of stamens and the style branches (**Figures 1E, F**, **2B**). An individual flower remained anthetic for three days; when the tepal lobes changed their colour to violet and dull black (**Figure 1A**, central flowers), the anthesis was considered to be over.

### Floral visitors

Flowers of *B. disticha* were visited by flies (Diptera), bees and bumblebees (Hymenoptera), butterflies and moths (Lepidoptera) and occasionally by orthopterans and spiders (Araneae). The floral visitors were predominantly recorded during day time; night visitors were rare, represented by nocturnal orthopterans only. The number and time of visits are summarized in **Table 1**.

**Table 1:** Numbers of visitors observed on flowers of *Burmannia disticha* (based on captured visitors, direct observations and video records).

Interval of observation		0:00–1:00	1:00–2:00	200–3:00	3:00–4:00	4:00–5:00	5:00–6:00	6:00–7:00	7:00–8:00	8:00–9:00	9:00–10:00	10:00–11:00	11:00–12:00	12:00–13:00	13:00–14:00	14:00–15:00	15:00–16:00	16:00–17:00	17:00–18:00	18:00–19:00	19:00–20:00	20:00–21:00	21:00–22:00	22:00–23:00	23:00–0:00
Duration of observations (min)		60	60	1	0	0	11	60	60	70	60	60	62	182	246	300	311	301	90	115	120	80	60	54	50
Diptera	Muscidae											2	2	2	2		1								
	Syrphidae										1														
Hymenoptera	*Coelioxys* sp. (Megachilidae)																1								
	*Bombus burmensis* (Apidae)												2		1	3	3								
Lepidoptera	Hesperiidae														3	3	2	2							
	Pyralidae																1	3							
	Satyridae													1			1								
Orthoptera																									1
Araneae												2	1												

Flies (Diptera) from the families Muscidae and Syrphidae were recorded. They visited the flowers from 9:00 to 16:00. The flies landed on the tepal lobes and apparently intended to reach the floral chamber with their proboscises, but could not penetrate beyond the stigmas (and under the outer tepal lobes if they visited flowers that were not yet fully open).

**Figure 3:**
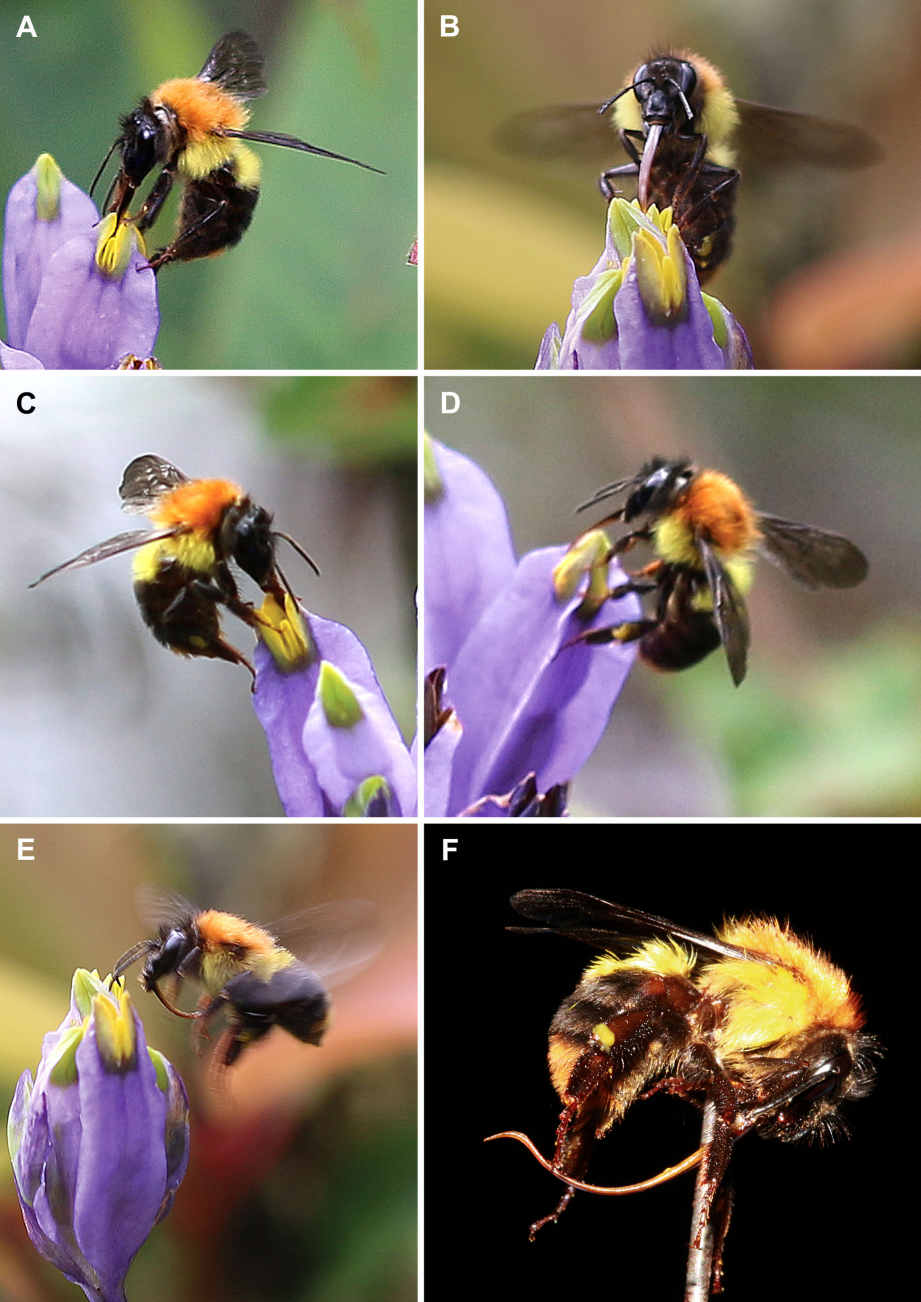
Bumblebees *Bombus burmensis* visited flowers of *Burmannia disticha*. **(A–E)** Insects during visitation. **(F)** Captured insect exhibiting length of proboscis.

Hymenopteran visitors were represented predominantly by bumblebees and also by a single bee. These insects were observed on the flowers from 11:00 to 16:00. They landed on the floral tube and tepal lobes and inserted their proboscises into the flower by bending the distal part of the transversally folded stigma down, accessing the floral chamber. Pollen attached to their heads is thus expected to be deposited on the brush-like papillae of the stigmas. All the nine observed bumblebees are members of the *Bombus trifasciatus* species complex, a group with complicated species delimitation (Hines and Williams, 2012). The captured insects are most close to *Bombus burmensis* Williams, 2020 (**Figure 3**). The captured bumblebees (seven individuals, see **Table 2**) have proboscises 7–11 mm long, which is long enough to reach nectar from the bottom of the floral chamber, whereas the recorded bee (belonging to the genus *Coelioxys* Latreille, 1809) has a proboscis about 3 mm long, i.e. significantly shorter than the floral tube in *B. disticha*.

**Table 2:** Numbers of pollen grains found in washings from flower visitors of *Burmannia disticha*.

Visitors		Number of investigated individuals	Total number of pollen grains		Number of pollen grains per individual		
			pollen of *Burmannia*	other pollen	pollen of *Burmannia* (mean ± SE)	other pollen (mean ± SE)	
Diptera	Muscidae	1	0	2	0	2
	Syrphidae	1	0	19	0	19
Hymenoptera	*Coelioxys* sp. (Megachilidae)	1	31	458	31	458
	*Bombus burmensis* (Apidae)	7	2556	6035	365 ± 117	862 ± 638
Lepidoptera	Hesperiidae	2	0	13	0	6.5 ± 0.5
	Pyralidae	2	2	13	1 ± 1	6.5 ± 1.5	
	Satyridae	1	0	24	0	24	
Orthoptera		1	0	0	0	0
Araneae		1	0	2	0	2

SE, standard error.

**Figure 4:**
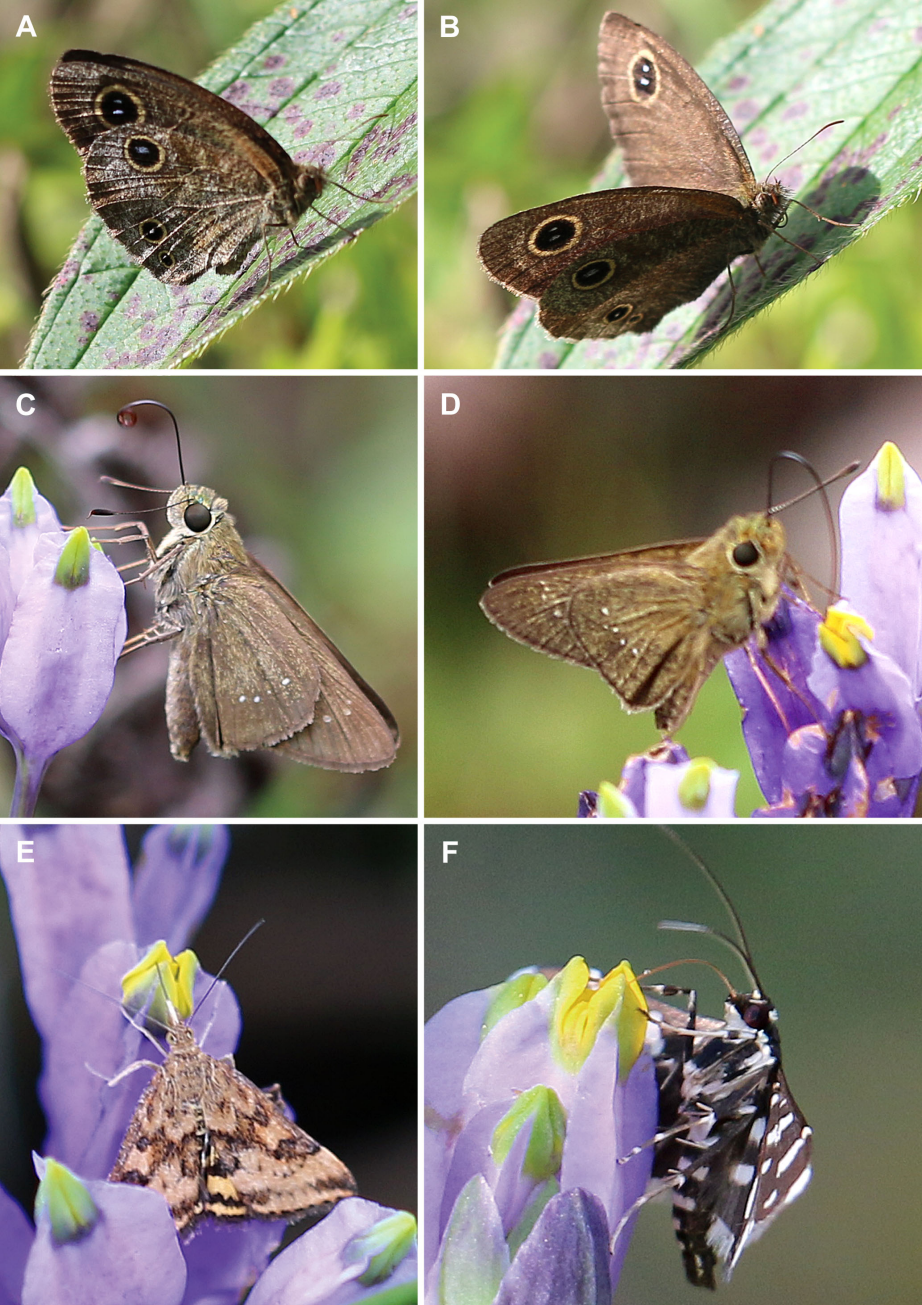
Butterflies and moths (Lepidoptera) visiting flowers of *Burmannia disticha*. **(A, B)** A butterfly belonging to Satyridae, right after visit of flowers of *B. disticha*. **(C, D)** Butterflies belonging to Hesperiidae during visitation. **(E, F)** Moths belonging to Pyralidae during visitation.

Lepidopterans were represented by butterflies from the families Hesperiidae and Satyridae, and moths belonging to Pyralidae. They visited flowers of *B. disticha* from 12:00 to 17:00 (**Figure 4**). Hesperiidae were the most frequent of them. The lepidopterans landed on the floral tube and tepal lobes and inserted their proboscises between the transverse folding of the stigma and the floral tube (slightly bending the stigma), i.e. in a manner similar to that of the hymenopterans. The lengths of the proboscises of captured insects were: 20–23 mm in Hesperiidae, about 6 mm in Pyralidae, and 7 mm in Satyridae, which allowed all these lepidopterans to reach the bottom of the floral chamber.

Orthopteran insects and spiders occasionally visited the flowers during day and night. All of them set on the outer surface of the floral tube and ovary never touching the tepal lobes or trying to get inside the floral chamber.

### Pollen grains on insect bodies, and the quantity of pollen and ovules in flowers

Among the visitors, pollen of *B. disticha* was detected only in the washings from the hymenopterans and the pyralid moths (**Table 2**). The largest number of pollen grains was detected on bumblebees: 365 pollen grains per insect in average. Pollen grains of other seed plants were detected on the bodies of dipterans, hymenopterans, lepidopterans and Araneae.

In *B. disticha*, number of pollen grains in one anther theca ranges from 1638 to 2563 (1990 in average). Number of ovules in one placenta ranged from 488 to 767 (582 in average). Pollen-ovule ratio for *B. disticha* was estimated as 6.84 (**Table 3**).

**Table 3:** Pollen-ovule ratio in *Burmannia disticha*.

Number of pollen grains in investigated thecae (listed in ascending order)		Number of ovules in investigated placentas (listed in ascending order)		
1638		488		
1814		517		
1929		535		
1959		565		
2036		620		
2563		767		
mean ± SE per theca	per flower	mean ± SE per placenta	per flower	Pollen-ovule ratio
1990 ± 313	11940	582 ± 101	1746	6.84

SE, standard error.

## Discussion

### *Burmannia disticha* shows entomophilous floral syndrome

The entire genus *Burmannia* is likely to possess a prominent entomophilous floral syndrome, as defined by Faegri and van der Pijl (1979). This idea has already been proposed for various species of the genus (Spitmann, 1975; Maas et al., 1986; Zhang and Saunders, 2000). Flowers of *Burmannia* are brightly coloured; in *B. disticha*, yellow tepal lobes contrast with the violet floral tube and ovary. The tepal lobes possibly serve as a visual cue signaling the entrance to the floral tube (where the nectar is presented), or imitating anthers for pollen-collecting insects. In particular, yellow coloration of the inner part of the floral display is known to be similar to the pollen coloration for Apoidea (Hymenoptera), where bees and bumblebees belong (Heuschen et al., 2005). It is likely that the flowers of *B. disticha* have no cues of any other types, since we found them to be odorless, at least to humans. According to Maas et al. (1986), there are no records of scented flowers in the genus; however, Zhang (1999) noted fragrance in flowers of *B. coelestis*. The other features of the entomophilous syndrome in *Burmannia* include adaxially papillose outer tepal lobes and nectar secretion via septal nectaries (e.g. Yudina et al., 2022). The presence of nectar was directly observed only in *B. lutescens* (Kato, 1996) and in *B. disticha* (present study). Thus, the flowers of *Burmannia* possess a primary attractant, i.e. a reward (nectar) and secondary attractants (coloration pattern and, in certain species, odor). In our study, attraction of visitors (insects) by the flowers of *B. disticha* is shown for the first time. The presence of floral visitors together with the modes of the flower-insect interactions reported here is in concordance with the idea of entomophily of this species.

### Flowers of *Burmannia disticha* are specialized for insects withlong proboscises

Flower of *B. disticha* exhibits the following traits that limit the access to the pollen, stigmas and nectar. The stamens and stigmas are enclosed in the floral chamber which is hardly accessible from the outside. The connectives are fused with the common style into a gynostegium (Yudina et al., 2022). The inner tepal lobes of the fully open flower possibly also contribute to the limited accessibility of the floral chamber, since they are arranged close to the gaps between the neighboring style branches. Then, the stigma is fan-shaped, so that the potential visitors (first of all, insects) are expected to be able to bend the stigma margin down and get inside the floral chamber via the gap between the floral tube, two stamens and the common style. Finally, the nectar is accumulated at the bottom of a long chamber, which further complicates access to this reward.

Two logical ways exist for the potential visitors to reach the nectar (apart from the nectar robbing): (1) by an animal small enough to enter the floral chamber; such an animal would be able to move within the chamber freely; (2) by an animal with a proboscis thin enough to enter the floral chamber and long enough to reach the bottom of the chamber. In our study, not a single tiny visitor was detected, and therefore the implementation of the first way has not been demonstrated. Among the recorded visitors, the bumblebees and the lepidopterans were observed to apparently get access to the floral chamber successfully. These insects penetrated the flower entrance with their proboscises, and most of them have the proboscises at least as long as the floral chamber. Thus, the second way of interaction between the flower and the visitor is likely to be employed by *B. disticha*.

In total, our study demonstrates that despite the “closed” appearance, the flower of *B. disticha* is well accessible for certain visitors, contrary to the views of e.g. Schoch (1920) who considered such a floral construction as an argument in favour of self-pollination.

### Bumblebees and lepidopterans are probable pollinators of *Burmannia disticha*

Among the hymenopterans, the bumblebees (*Bombus burmensis*) and a bee (*Coelioxys* sp.) visited the flowers of *B. disticha*. They inserted their proboscises inside the floral tube. At the same time, all of them bore pollen grains of *B. disticha*, and the bumblebees bore it in large amounts. Our results thus suggest that the bumblebees and bees act as pollinators of *B. disticha*. Since the bumblebees had proboscises 7–11 mm long, they were likely able to suck the nectar from the bottom of the floral chamber that was estimated as 5.3–8.1 mm deep. On the other hand, they could also possibly collect pollen, because bumblebees are known to use both honey and pollen for food and storage (Michener, 2007; Williams, 2020) and were even shown to have preferences for two-coloured flowers with a centre colour similar to that of pollen (Heuschen et al., 2005). The bee, in contrast, had a short proboscis about 3 mm long, probably reaching only anthers but not nectar. *Coelioxys* is a genus of cleptoparasitic bees, i.e. they lay their eggs in the nests of host species. Their hosts are other species of bees, mainly belonging to *Megachile* Latreille, 1802 and various Apidae; the parasite larva feeds on the food that had been provided for the host larvae (Michener, 2007). Accordingly, adults of *Coelioxys* bees lack any pollen-manipulating or pollen-carrying structures, and only forage for their own needs as the brood is cared for by the host species (Martin et al., 2000). Parasitic bees usually take only nectar from flowers, which they use as their own food. They only carry pollen that deposits passively on them. Parasitic bees are often not very hairy and thus probably play a less significant role in pollination than the pollen-collecting bees (as well as hairy bees) do (Michener, 2007). Thus, the bee captured on *B. disticha* possibly visited the flowers accidentally, while browsing bright flowers for foraging, and cannot be considered as a regular pollinator of *B. disticha*.

Among the lepidopterans, another group of frequent visitors of *B. disticha* in our study, only Pyralidae carried pollen of *B. disticha*, and only in a small amount. Though, the amount of pollen in the washings from the bodies of lepidopterans was possibly underestimated: numerous scales from the lepidopteran wings appeared in the washings together with pollen, which complicated detection of the pollen grains. All the recorded lepidopterans (especially Hesperiidae) had proboscises long enough to reach the nectar. The majority of lepidopterans (including the visitors of *B. disticha*) are anthophilous in adult stages, and nectar is known to be their principal nutrient source (Gilbert and Singer, 1975; Krenn, 2010), whereas for only a few lepidopteran species adaptations to pollen-feeding have been described (Romeis et al., 2005). At the same time, the majority of species likely benefit from pollen-derived nutrients when consuming pollen-contaminated nectar (Baker and Baker, 1975; Romeis et al., 2005). Pollen of *Burmannia* (including *B. disticha*) was indicated as starch-rich but lipid-poor (Zhang, 1999; Zhang and Saunders, 2000), which is typical of plants pollinated by Lepidoptera (Baker and Baker, 1983). Although Zhang and Saunders (2000) stated that “the small size of *Burmannia* flowers and the inaccessible anthers preclude wind-pollination or pollination by Lepidoptera or birds”, our data allow to refute this idea at least regarding Lepidoptera. Considering all the evidence, we argue that lepidopterans might play a significant role in pollination of *B. disticha*.

Although flies were relatively frequent visitors of flowers of *B. disticha*, they did not get access to the floral tube and therefore did not reach anthers and stigmas. Accordingly, pollen of *B. disticha* was not found on their bodies (though there was pollen of other flowering plants). They might have been attracted by the yellow tepal lobes resembling anthers, as pollen is included in the diet of some flies, especially Sirphidae. Spiders and orthopterans visited flowers occasionally and bore virtually no pollen grains on their bodies. All these groups of visitors evidently do not take part in pollination of *B. disticha*; instead, the spiders may be floral visitor predators, and the orthopterans may be florivores (but we have not detected any traces of florivory).

### *Burmannia disticha* is xenogamous with some attributes of autogamy

Three types of mating systems are distinguished in angiosperms according to the mode of gene transfer from one generation to the next through sexual reproduction: xenogamy, geitonogamy and autogamy (including cleistogamy) (Cardoso et al., 2018). Asexual reproductive systems, i.e. apomixis, which is currently known for a single species of *Burmannia* (Ernst, 1909), cannot be excluded for *B. disticha*, but unlikely occur in this species given the open anthetic flowers and visitations by insects.

Geitonogamy (which is equal to autogamy from the genetic and evolutionary viewpoint, but resembles xenogamy with respect to the pollination mechanism) is unlikely for the entire genus *Burmannia* for the reason of shoot structure in the genus. Indeed, in *B. disticha*, an individual develops a single inflorescence, and within an inflorescence the flowers open sequentially and not more than two of them bloom simultaneously. The cymose inflorescence structure can therefore be viewed as an adaptation against fertilization of an individual’s ovules by its own pollen (that would be performed in case of geitonogamy).

As demonstrated here, *B. disticha* is characterized by an entomophilous floral syndrome and shows adaptations for pollination by insects with long proboscises. Moreover, we revealed pollen transport by insects (predominantly bumblebees) in this species. Based on these findings, we conclude that *B. disticha* possesses a set of adaptations to xenogamy.

It should be taken into account that entomophily does not necessarily preclude from autogamous pollen transfer, i.e. the so-called facilitated autogamy (Lloyd, 1992; Cardoso et al., 2018). In *B. disticha*, this mechanism is possible, because the insects are expected to touch both stigmas and anthers of the same flower. However, facilitated autogamy was argued to be only rarely beneficial, being mainly a by-product of adaptations to cross-pollination (Lloyd, 1992). Therefore, entomophily can be considered a solid indicator of a xenogamous mating system.

Pollen-ovule ratio estimated in this study for *B. disticha* (6.84) evidences in favor of cleistogamy or obligate autogamy according to the analysis by Cruden (1977), whereas the ratios found by him in xenogamous angiosperms were above 20. In line with this finding, self-pollination as well as self-compatibility was assumed previously for a number species of *Burmannia* including *B. disticha* (Schoch, 1920; Fryxell, 1957; Wood, 1983; Zhang, 1999). Thus, a certain contribution of autogamy to the mating system of *B. disticha* is likely to take place. This inference does not contradict the idea of predominance of xenogamy in this species. With respect to the pollen-ovule ratio, as it has already been argued by Yudina et al. (2021) for some other genera of Dioscoreales (*Tacca* J.R.Forst. & G.Forst., Taccaceae, and *Thismia* Griff., Thismiaceae), the low values indicate high efficiency of pollen transfer that is not necessarily facilitated by autogamy. Moreover, a possibility of a versatile pollination strategy in a species should be considered. Comparing our results with the observations by Zhang (1999), one can infer that both insect-mediated cross-pollination and autogamous seed set via cleistogamy occur in *B. disticha*, possibly employed by different populations or depending on environmental conditions.

## Conclusions

We provided results of the first comprehensive study of pollination biology of a species of Burmanniaceae based on field observations in natural habitat of the species. We demonstrated that *Burmannia disticha* not only possesses entomophilous syndrome that includes perianth opening, but also actually attracts visitors (insects) belonging to groups generally known to act as pollen vectors. As our data show, the closed structure of flower does not prevent insects from accessing pollen, stigmas and nectar. We therefore characterize the flowers of *B. disticha* as chasmogamous.

Consistently with the size, shape and accessibility of the nectar-containing floral tube, the flowers of *B. disticha* were visited by insects with long proboscises. Among them, we consider bumblebees (*Bombus burmensis*) to be the most effective pollinators based on their visit frequency and abundance of *Burmannia* pollen on their bodies. In addition, lepidopterans potentially play a significant role in pollination of *B. disticha*.

Our results suggest that *B. disticha* possesses a set of adaptations to xenogamous pollination with nectar-consuming insects as pollen vectors. At the same time, evidences of autogamy in this species exist, including the pollen-ovule ratio estimated in our study and seed set in a closed flower observed by Zhang (1999). We suppose that *B. disticha* shows a labile pollination strategy with xenogamy as the primary mating system. The results of our study are to be further corroborated by experiments of artificial pollination.

Data on pollination of the fully autotrophic *B. disticha* are important for further understanding of reproductive diversity in *Burmannia*, and in particular for comparison with its species having other modes of nutrition. Observations on the fully mycoheterotrophic *B. lutescens* revealed visitations by dipterans (Kato, 1996; Momose et al., 1998), which suggests a different pollination syndrome.

## Data availability statement

The original contributions presented in the study are included in the article/supplementary material. Further inquiries can be directed to the corresponding author.

## Author contributions

NV was involved in conceptualization, fieldwork, sectioning, SEM studies, microscopy, assembled the figures and drafted the manuscript. MN was involved in preparation of the original draft and figures, conceptualization and interpretation. All the authors reviewed drafts of the paper, and approved the final version. All authors contributed to the article and approved the submitted version.

## References

[B1] Baker H. G. Baker I. (1975). “Studies of nectar-constitution and pollinator-plant coevolution,” in Coevolution of animals and plants. Eds. Gilbert L. E. Raven P. H. (Austin: University of Texas Press), 100–140. doi: 10.7560/710313-007

[B2] Baker H. G. Baker I. (1979). Starch in angiosperm pollen grains and its evolutionary significance. Am. J. Bot. 66, 591–600. doi: 10.1002/j.1537-2197.1979.tb06262.x

[B3] Baker H. G. Baker I. (1983). “Some evolutionary and taxonomic implications of variation in the chemical reserves of pollen,” in Pollen: biology and implications for plant breeding. Eds. Mulcahy D. L. Ottaviano E. (New York: Elsevier Biomedical), 43–52.

[B4] Bui H. Q. Tran T. B. Eum S. Do V. H. Nguyen K. S. Le N. H.. (2020). Towards a floristic inventory of Bat Xat Nature Reserve, Vietnam: Thirteen new national records of vascular plants. Wulfenia 27, 233–250.

[B5] Caddick L. R. Rudall P. J. Wilkin P. (2000). Floral morphology and development in Dioscoreales. Feddes Repert. 111, 189–230. doi: 10.1002/fedr.20001110313

[B6] Cardoso J. C. F. Viana M. L. Matias R. Furtado M. T. Caetano A. P. D. S. Consolaro H.. (2018). Towards a unified terminology for angiosperm reproductive systems. Acta Bot. Bras. 32, 329–348. doi: 10.1590/0102-33062018abb0124

[B7] Cruden R. W. (1977). Pollen-ovule ratios: a conservative indicator of breeding systems in flowering plants. Evolution 31, 32–46. doi: 10.2307/2407542 28567723

[B8] Dang V. S. Tagane S. Toyama H. Yahara T. Naiki A. Quan N. H.. (2015). A new record of *Burmannia championii* Thwaites (Burmanniaceae) from Southern Vietnam. J. Biotechnol. 13, 1393–1396.

[B9] Ernst A. (1909). Apogamie bei *Burmannia coelestis* Don. Ber. Deu. Bot. Ges. 27, 157–168. doi: 10.1111/j.1438-8677.1909.tb06782.x

[B10] Ernst A. Bernard C. (1912). Beiträge zur Kenntnis der Saprophyten Javas. IX. Entwicklungsgeschichte des Embryosacks und des Embryos von *Burmannia candida* Engl. und *B. championii* Thw. Ann. Jard. Bot. Buitenzorg 25, 161–188.

[B11] ESRL (2023) Earth System Research Laboratory, Global Monitoring Division, Global Radiation Group, Solar Calculator. Available at: http://www.esrl.noaa.gov/gmd/grad/solcalc (Accessed May 25, 2023).

[B12] Faegri K. van der Pijl L. (1979). The principles of pollination ecology. 3rd ed (New York: Pergamon Press).

[B13] Francis D. Mohan V. Venugopal D. K. Nampy S. (2021). *Burmannia munnarensis* (Burmanniaceae) a new species and rediscovery of *B. indica* after 110 years from southern Western Ghats, Kerala, India. Phytotaxa 507, 105–112. doi: 10.11646/phytotaxa.507.1.6

[B14] Fryxell P. A. (1957). Mode of reproduction of higher plants. Bot. Rev. 23, 135–233. doi: 10.1007/BF02869758

[B15] Gilbert L. E. Singer M. C. (1975). Butterfly ecology. Annu. Rev. Ecol. Syst. 6, 365–397. doi: 10.1146/annurev.es.06.110175.002053

[B16] Govaerts R. Saunders R. M. K. Maas-van de Kamer H. Maas-van de Kamer P. Zhang D. X. (2022) World checklist of Burmanniaceae (Royal Botanic Gardens, Kew). Available at: http://wcsp.science.kew.org/ (Accessed 29 September, 2022).

[B17] Heuschen B. Gumbert A. Lunau K. (2005). A generalised mimicry system involving angiosperm flower colour, pollen and bumblebees’ innate colour preferences. Plant Syst. Evol. 252, 121–137. doi: 10.1007/s00606-004-0249-5

[B18] Hines H. M. Williams P. H. (2012). Mimetic colour pattern evolution in the highly polymorphic *Bombus trifasciatus* (Hymenoptera: Apidae) species complex and its comimics. Zool. J. Linn. Soc. 166, 805–826. doi: 10.1111/j.1096-3642.2012.00861.x

[B19] Hsieh T. H. Ohashi H. (2000). A new record of *Burmannia championii* Thwaites (Burmanniaceae) in Taiwan. Taiwania 45, 346–350. doi: 10.6165/tai.2000.45(4).346

[B20] Jonker F. P. (1938). A monograph of the Burmanniaceae (Utrecht: Mededeelingen van het Botanisch Museum en Herbarium van de Rijks Universiteit de Utrecht).

[B21] Jonker F. P. (1948). “Burmanniaceae,” in Flora Malesiana I, vol. 4) . Ed. van Steenis C. G. G. J. (Jakarta and Leiden: Noordhoff), 12–26.

[B22] Kato M. (1996). Plant-pollinator interactions in the understory of a lowland mixed dipterocarp forest in Sarawak. Am. J. Bot. 83, 732–743. doi: 10.1002/j.1537-2197.1996.tb12762.x

[B23] Krenn H. W. (2010). Feeding mechanisms of adult Lepidoptera: structure, function, and evolution of the mouthparts. Annu. Rev. Entomol. 55, 307–327. doi: 10.1146/annurev-ento-112408-085338 19961330 PMC4040413

[B24] Li X. Zhang K. Qian X. Wu M. Zhang D. (2020). *Burmannia decurrens* (Burmanniaceae), a new mycoheterotrophic species from southwestern Guangdong, China. Nordic J. Bot. 38, e02718. doi: 10.1111/njb.02718

[B25] Lloyd D. G. (1992). Self- and cross-fertilization in plants. II. The selection of self-fertilization. Int. J. Plant Sci. 153, 370–380. doi: 10.1086/297041

[B26] Maas P. J. M. Maas-van de Kamer H. van Benthem J. Snelder H. C. M. Rubsamen T. (1986). Burmanniaceae. Flora Neotropica Monograph 42, 1–189.

[B27] Martin H. J.. (2000) Wildbienen. Available at: https://www.wildbienen.de (Accessed 4 October, 2022).

[B28] Merckx V. S. F. T. Freudenstein J. V. Kissling J. Christenhusz M. J. M. Stotler R. E. Crandall-Stotler B.. (2013). “Taxonomy and classification,” in Mycoheterotrophy: The biology of plants living on fungi. Ed. Merckx V. S. F. T. (New York: Springer Science+Business Media), 19–101. doi: 10.1007/978-1-4614-5209-6_2

[B29] Merckx V. S. F. T. Stöckel M. Fleischmann A. Bruns T. D. Gebauer G. (2010). ^15^N and ^13^C natural abundance of two mycoheterotrophic and a putative partially mycoheterotrophic species associated with arbuscular mycorrhizal fungi. New Phytol. 188, 590–596. doi: 10.1111/j.1469-8137.2010.03365.x 20618915

[B30] Mesta D. K. Hegde H. V. Upadhya V. Kholkute S. D. (2011). *Burmannia championii* Thwaites (Dioscoreales: Burmanniaceae), a new addition to the flora of Karnataka. J. Threatened Taxa 3, 1465–1468. doi: 10.11609/JoTT.o2495.1465-8

[B31] Michener C. D. (2007). The bees of the World. 2nd ed (Baltimore, Maryland: The Johns Hopkins University Press).

[B32] Momose K. Yumoto T. Nagamitsu T. Kato M. Nagamasu H. Sakai S.. (1998). Pollination biology in a lowland dipterocarp forest in Sarawak Malaysia. I. Characteristics of the plant-pollinator community in a lowland dipterocarp forest. Am. J. Bot. 85, 1477–1501. doi: 10.2307/2446404 21684899

[B33] Nuraliev M. S. Yudina S. V. Truong B. V. Dang V. S. Kopylov-Guskov Y. O. Lyskov D. F.. (2022). A checklist of Burmanniaceae in Eastern Indochina with a new record from Vietnam, *Burmannia itoana* . Phytotaxa 544, 61–70. doi: 10.11646/phytotaxa.544.1.5

[B34] Nuraliev M. S. Zhang D. Kuznetsov A. N. Kuznetsova S. P. (2018). Two new records of non-photosynthetic *Burmannia* species (Burmanniaceae) from Laos and Vietnam. Wulfenia 25, 52–56.

[B35] Romeis J. Städler E. Wäckers F. L. Bruin J. (2005). “Nectar- and pollen-feeding by adult herbivorous insects,” in Plant-provided food for carnivorous insects: a protective mutualism and its applications. Eds. Wäckers F. L. van Rijn P. C. J. (Cambridge: Cambridge University Press), 178–220.

[B36] Saunders R. M. K. (1996). The occurrence and taxonomic relationships of *Burmannia wallichii* (Burmanniaceae) in Malesia. Blumea 41, 333–337.

[B37] Schoch M. (1920). Entwicklungsgeschichtlich-cytologische Untersuchungen über die Pollenbildung und Bestäubung bei einigen Burmannia-Arten (Zürich: Institut für Allgemeine Botanik, Universität Zürich).

[B38] Spitmann A. (1975). Entwicklungsgeschichtliche Untersuchungen an Burmannia stuebelii Hieron. et Schltr. (Bochum: Universität Bochum).

[B39] Suetsugu K. Kawakita A. Kato M. (2014). Evidence for specificity to *Glomus* group Ab in two Asian mycoheterotrophic *Burmannia* species. Plant Spec. Biol. 29, 57–64. doi: 10.1111/j.1442-1984.2012.00387.x

[B40] Suetsugu K. Sando T. (2017). First record of *Burmannia cochinchinensis* Gagnep. (Burmanniaceae) from Kabin Buri District, Prachinburi, Thailand. Check List 13, 1–3. doi: 10.15560/13.2.2070

[B41] Tsukaya H. Darnaedi D. (2012). *Burmannia bengkuluensis* sp. nov. (Burmanniaceae) from Sumatra. Nordic J. Bot. 30, 159–162. doi: 10.1111/j.1756-1051.2011.01360.x

[B42] Williams P. H. (2020). *Bombus burmensis* replaces *B. malaisei* (Skorikov). J. Nat. Hist. 53, 2737–2738. doi: 10.1080/00222933.2020.1732491

[B43] Wood C. E. (1983). The genera of Burmanniaceae in the southeastern United States. J. Arnold Arbor. 64, 293–307. doi: 10.5962/p.324744

[B44] Wu D. Zhang D. Saunders R. M. K. (2010). “Burmanniaceae,” in Flora of China. Eds. Wu Z. Y. Raven P. H. Hong D. Y. (Beijing and St. Louis: Hong Science Press and Missouri Botanical Garden), 121–124.

[B45] Yudina S. V. Kocyan A. Truong B. V. Vislobokov N. A. Lyskov D. F. Nuraliev M. S.. (2022). Structure and development of flowers and inflorescences in *Burmannia* (Burmanniaceae, Dioscoreales). Front. Plant Sci. 13. doi: 10.3389/fpls.2022.849276 PMC8971816 35371135

[B46] Yudina S. V. Vislobokov N. A. Nuraliev M. S. (2021). Evidences of a mixed pollination strategy in Vietnamese species of *Thismia* (Thismiaceae: Dioscoreales). Wulfenia 28, 109–128.

[B47] Zhang D. (1999). Systematics of Burmannia L. (Burmanniaceae) in the Old World (Hong Kong, Pokfulam: University of Hong Kong). doi: 10.5353/th_b3123973

[B48] Zhang D. Saunders R. M. K. (2000). Reproductive biology of a mycoheterotrophic species, *Burmannia wallichii* (Burmanniaceae). Bot. J. Linn. Soc. 132, 359–367. doi: 10.1111/j.1095-8339.2000.tb01217.x

